# Inflammation of Vein

**Published:** 1828-12

**Authors:** 


### INFLAMMATION OF VEIN.

### *Inflammation of the Vein: Purulent Depôts in and about the Shoulder and Knee-Joint.*

Alexander Dobie, æt. twenty-eight, a stout and apparently healthy young man, was admitted into St. George's Hospital, at half-past three o'clock in the afternoon of the 14th September, with a tumor (apparently strangulated hernia) in the right groin. Five years before his admission, a second tumor made its appearance, which afterwards often came down, but could always be returned. Two or three years subsequently to its first appearance, the hernia became strangulated, but at the end of three days was reduced by a surgeon; since which occurrence he had generally worn a truss. While digging in a garden the day before admission, the hernia came down, and resisted all attempts at reduction.

The man was of a remarkably captious and intractable temper, and prevented Mr. Keate from employing the taxis long or efficiently.

He was bled to thirty ounces; the taxis thrice tried, without effect; sickness supervened; and under these circumstances Mr. K. proposed the operation to the patient, who absolutely refused to undergo it. Calomel and extract of Hyosciamus, followed by tobacco enema, were ordered; and next day the tenderness of abdomen had subsided, whilst the tumor still remained. With the aid of a senna draught, free evacuations from the intestinal canal took place on the two succeeding days; the local pain and tenderness quickly passed away; the hernia, though it never decidedly and suddenly "went up," progressively diminished in size, and ceased to be an object of attention.

A new train of symptoms now arose: symptoms attended, in this hospital at least, with uniformly disastrous and fatal results.

The wound which had been made by the lancet in bleeding inflamed, but at first was considered a common sore arm. The patient being feverish, was ordered, on the 22d, salines, with antimonial wine, and a purgative.

On the 25th, however, the inflammation had increased; the arm was swollen from the elbow to the shoulder; a blush of erythema suffused the skin; the vein was thickened above the puncture; and matter could be pressed, to some slight extent, from the latter. No red line in the course of the vein was noticed at this or any subsequent time. The skin was hot; the tongue moist and coated; pulse eighty-six; bowels open; no delirium, and little alteration of manner.

**Pulv. Ip. grs. xx.; Ant. Tart. gr. i. pro emetico.—Hyd. Subm. grs. v.; Opii gr. iss. post horas tres.—Liq. Ammon. Acet.; Aq. distil. āā ℥vj.; Syr. ℥i. quartis horis.**

On the 26th, he complained of shooting pain about the shoulder, which increased next day; in addition to which, matter seemed forming under the fascia at the bend of the arm. In the afternoon he had a rigor. At five P.M. an incision was made at the inside of the arm, opposite the inner condyle, but nothing was discovered. No relief ensued, and the morning of the 28th was ushered in by another fit of shivering, followed by reaction and sweating. The arm was now beginning to improve, and never after gave much inconvenience.

On the 30th, he was ordered Camphor Mixture, with the Liquor Ammoniæ Acetatis. A rigor was experienced in the evening. He passed an indifferent night, and was worse on the 31st. The arm was kept constantly enveloped in a linseed poultice, and placed in the semi-bent position.

On the 3d of November, the arm was so well that the common erysipelas ointment was substituted for the linseed poultice; but on the 4th the patient all at once complained of pain in the right knee, which, as well as the ham and calf of the leg, was swollen and tender upon pressure. A lotion was applied, and a mixture taken, consisting of—

**Haust. Salin. ℥iss.; Tinct. Humuli ʒi.; Vin. Ant. Tart. m. xx.; Tinct. Opii m. vi. quartis horis.**

On the next day, the swelling and pain had increased, attended with some slight dyspnœa and cough. Ten leeches were applied, and succeeded on the 6th by eighteen more.

On the 7th, there was a pause in the local symptoms, but the voice was remarkably husky; the breathing not natural nor free.

On the 8th, no pain was experienced in the knee, but decided fluctuation was perceived both in and about the joint. Salines, with opium and tincture of bark, were given. Next day the parts were hot and tender, in consequence of which ten leeches were applied, and a blister placed both above and below the knee on the 11th. The swelling, notwithstanding, extended; the fluctuation became more distinct; diarrhœa set in; and the system was evidently suffering severely.

On the 22d, an opening was made at the inferior part, and outside of the thigh, and five ounces of pus, or thereabouts, let out. This incision in the thigh was followed by another in the shoulder, two or three days afterwards: from the latter was evacuated an ounce of fetid pus. On the 27th, the opening in the thigh was enlarged, and next day another incision had recourse to on the inside of the knee. On the 31st, it was once more necessary to employ the lancet in the calf.

The patient expired on the 2d November.

*Dissection.*—State of the veins of the arm: All trace of inflammation had passed from without, and the puncture was healed. The median basilic had been wounded, which vein, in conjunction with one or two branches of the basilic itself about the inner condyle, was thickened in its coats, and obliterated in its cavity. Some half-inch, or inch, above the puncture, the median basilic was pervious again, and so continued for two or three inches, when again its calibre was diminished, and its cavity choked up. This state of things continued as high as the junction of the common basilic and subscapular, that junction which constitutes the axillary trunk. Here the inflammation ceased; and the axillary was as free, as healthy, and capacious as ever. The cellular membrane, in the course of the brachial vessels, was condensed, and the vessels themselves in some degree matted together.

State of the joints: Under the deltoid was one *foyer* of dark and semiputrid looking pus. The bone was bare; the capsule of the shoulder-joint was gone, destroyed; the articulating cartilages destroyed also: in short, the most extensive mischief had occurred both within and without the joint.

For the lower half of the thigh, the femur was literally insulated from the soft parts round by one collection of putrid matter: periosteum, muscle, all were involved alike. The capsule, as in the shoulder, was no longer to be seen, at least in its upper part; the matter could be poured from the thigh into the knee-joint, or from that again into the thigh, with perfect facility; the cartilages were almost entirely destroyed, and the limb presented a shocking appearance.

The viscera shewed nothing remarkable. Head not examined.*

* Medical Gazette.

